# Impact of rheumatoid arthritis on Alzheimer’s disease: A two-sample bidirectional Mendelian randomization study

**DOI:** 10.1097/MD.0000000000046518

**Published:** 2025-12-12

**Authors:** Songxin Zhong, Changqiang Feng, Shizhan Li, Yanni Lin, Jianqing Zhu, Xia Liang, Chao Qin

**Affiliations:** a Department of Neurology, The First Affiliated Hospital of Guangxi Medical University, Nanning, China; b Department of Neurology, The First People’s Hospital of Yulin Affiliated to Guangxi Medical University, Yulin, China.

**Keywords:** Alzheimer disease, causal relationship, Mendelian randomization, rheumatoid arthritis

## Abstract

Controversial relationship of rheumatoid arthritis (RA) with Alzheimer disease (AD) risk has been reported in previous research. However, epidemiological studies are susceptible to confounding factors and reverse causality. This study aimed to explore the causal relationship between RA and AD by using a 2-sample bidirectional Mendelian randomization (MR) study. Genetic data for RA and AD were extracted from published genome-wide association study databases and the FinnGen project. The primary statistical method was inverse variance weighted, which was supplemented by MR Egger regression, weighted median, simple mode, and weighted mode methods. In addition, Cochran *Q* test, MR Egger intercept, and MR-PRESSO global test were used to detect heterogeneity and pleiotropy. Further sensitivity analyses were conducted using leave-one-out method and funnel plots, to evaluate the robustness of the results. Genetically predicted RA had a positive casual effect on the risk of AD development (inverse variance weighted odds ratio [OR] = 1.062, 95% confidence interval [CI] = 1.019–1.107, *P* = .004; weighted median OR = 1.073, 95% CI = 1.021–1.126, *P* = .005; weighted mode OR = 1.077, 95% CI = 1.025–1.131, *P* = .007). Notably, reverse MR analysis indicated no significant effect of AD on RA (all *P* > .05). No pleiotropy or heterogeneity was identified in the bidirectional MR analysis. And leave-one-out analysis and funnel plots confirmed the robustness and reliability of the findings. This study provides new evidence for the causal relationship between RA and the increased risk of AD. Early screening of cognitive function in patients with RA may be beneficial in preventing future AD progression.

## 1. Introduction

Rheumatoid arthritis (RA) is a complex chronic autoimmune disease characterized primarily by joint inflammation, pain, stiffness, and functional impairment that significantly affect patients’ quality of life.^[1,2]^ Patients with RA not only face joint damage, but may also be accompanied by other systemic diseases, including cardiovascular, lung and neurological diseases.^[3,4]^ In particular, many studies have shown an association between RA and dementia.^[5–7]^ Alzheimer disease (AD), the most common type of dementia, is a progressive neurodegenerative disease that leads to gradual loss of cognitive function.^[8]^ Currently, an estimated 6.9 million Americans over the age of 65 suffer from AD, and this number may increase to 13.8 million by 2060.^[9]^ The characteristic changes of AD are amyloid beta (Aβ) deposits and neurofibrillary tangles.^[10]^ Further research has revealed that inflammation is also closely related to the occurrence and development of AD.^[11]^ Since there is no effective therapeutic drug to prevent the occurrence or reverse the progression of AD, early prevention of AD is particularly critical.

In recent years, research has explored the potential connections between RA and AD. The chronic inflammatory response and overreaction of the immune system are common features of both RA and AD.^[12,13]^ Overall, RA patients seem to have a higher risk of developing AD than the general population.^[14–16]^ For example, a cross-sectional secondary analysis based on Medicare Current Beneficiary Survey data showed that RA was significantly associated with a higher risk of AD.^[17]^ Another population-based, retrospective cohort study also revealed a correlation between RA and an increased risk of AD (adjusted hazard ratio: 1.37; 95% confidence interval [CI]: 1.04–1.81).^[18]^ Besides, cox regression models using the UK database demonstrated that the overall incidence of AD was higher in patients with RA than in controls.^[19]^ However, some epidemiological studies have indicated a negative correlation between RA and AD.^[20,21]^ This negative association may be affected by confounding factors, as studies have shown that RA therapeutic drugs such as tumor necrosis factor (TNF) inhibitors can reduce the incidence of dementia in patients with RA.^[22,23]^ Due to the ubiquitous challenges posed by confounding factors, reverse causality, and selection bias in observational studies, exploration of causal relationships between RA and AD is often severely constrained.

As RA and AD are both key issues affecting the elderly, efforts to shed light on their possible relationship will bring great benefits to a large part of elderly population. Mendelian randomization (MR) study is rooted in the vast data samples accumulated from genome-wide association study (GWAS), which is an innovative and alternative methodology for exploring causality in epidemiological research. This approach ingeniously utilizes genetic variations as naturally occurring random experimental tools, enabling precise exploration of potential causal links between risk factors and disease states.^[24]^ Over the last few years, many MR studies have uncovered potential causal links between AD and various conditions, living habits, as well as environmental exposures.^[25,26]^ Furthermore, several MR studies have tried to explain the causal relationship between RA and AD, resulting in inconsistent conclusions.^[27,28]^ Given the availability of updated and larger GWAS data, we now possess more evidence to deeply explore their causal relationship and strive to minimize bias to the greatest extent possible.

## 2. Materials and methods

### 2.1. Study design and data resource

**Figure 1.**
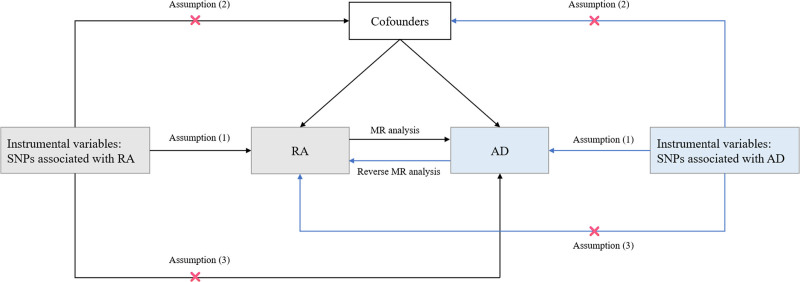
Flow chart of the 2-sample bidirectional MR study: assumption (1), instrumental variables must be strongly correlated with the exposure; assumption (2), instrumental variables must not be related to any confounding factors affecting the outcome; assumption (3), instrumental variables can only impact the outcome through the exposure. RA = rheumatoid arthritis, AD = Alzheimer disease, SNP = single nucleotide polymorphism, MR = Mendelian randomization.

A bidirectional 2-sample MR study was performed to assess the causal relationship between RA and AD in the European population. MR analysis used single nucleotide polymorphism (SNP) as instrumental variables and must meet the following 3 assumptions: instrumental variables must be strongly correlated with the exposure; instrumental variables must not be related to any confounding factors affecting the outcome; and instrumental variables can only impact the outcome through the exposure (Fig. 1). The report of this study followed the STROBE-MR reporting guidelines.^[29]^

To avoid sample overlap, RA and AD genetic data were obtained from 2 different GWAS datasets. GWAS summary statistics for RA were sourced from a cross-population atlas of genetic associations for 220 human phenotypes, including 8255 patients and 409,001 controls of European ancestry.^[30]^ Summary statistics for AD were extracted from the FinnGen database published in 2024, which includes 11,755 patients and 441,978 controls.^[31]^ As this MR study used publicly available data, no ethical approval or informed consent was required.

### 2.2. Instrumental variables selection

Based on the 3 assumptions, we first extracted SNP that were strongly related to exposure at a genome-wide significance level of *P* < 5 × 10^‐8^. Linkage disequilibrium clumping with *r*^2^ < 0.001 and kb = 10,000 was performed to exclude SNP with strong linkage disequilibrium. Furthermore, SNP with *F*-statistics <10 were eliminated to minimize the influence of weak instrumental bias.^[32]^ Subsequently, we employed the LDtrait tool (https://ldlink.nih.gov/?tab=ldtrait) to identify and eliminate SNP associated with potential confounders. Finally, we harmonized the exposure and outcome datasets to ensure the consistency of allele effects. Before performing MR analysis, MR-PRESSO analysis was conducted to identify and remove outliers with potential pleiotropic effects.^[33]^

### 2.3. MR analysis

The inverse variance weighted (IVW) method was considered to be the most stable and accurate causal relationship method and was usually used as the preferred statistical method for MR analysis. In addition, 4 complementary methods, including MR Egger, weighted median, simple mode, and weighted mode, were also used to infer causality. The MR Egger method can evaluate whether exposure affects the outcome when all instrumental variables are assumed to be invalid.^[34]^ When 50% of the instrumental variables are invalid, the weighted median method still provides a reliable causal estimation.^[35]^ The simple mode method and the weighted mode method collect SNP with similar causal effects and provide causal effect estimates for most SNP, while the weighted mode method also carries out weighted processing according to the inverse variance of SNP effect results.^[26]^ The MR results were quantified using the odds ratio (OR) and 95% CI, with statistical significance set at *P* < .05. Specifically, the causal association was considered meaningful when the IVW result was significant and all 5 methods revealed effects in the same direction.

### 2.4. Sensitivity analysis

Sensitivity analysis was conducted to evaluate the robustness of the MR results. Cochran *Q* test was used to evaluate the heterogeneity among instrumental variables. The MR Egger intercept test and MR-PRESSO Global test were performed to assess potential pleiotropy. A *P*-value < .05 indicated the existence of heterogeneity or pleiotropy. Furthermore, a leave-one-out analysis was performed to investigate whether the causal relationship was driven by a single SNP. The symmetry of the funnel plots could further strengthen the reliability of causal association. All statistical analyses were performed using the TwoSampleMR (version 0.6.1; University of Bristol, Bristol, UK) and MR-PRESSO packages (version 1.0; Ron Do Laboratory, New York) in R software (version 4.3.3; University of Bristol, Bristol, UK).

## 3. Results

### 3.1. Determination of instrumental variables

According to rigorous screening criteria, we identified 22 SNPs to serve as instrumental variables for RA in MR analysis and 19 SNPs to serve as instrumental variables for AD in reverse MR analysis. All SNPs demonstrated *F*-statistics >10, indicating no potential weak instrumental variables. Detailed information about the SNPs is available in Table S1, Supplemental Digital Content, https://links.lww.com/MD/Q888.

### 3.2. Casual effect of RA on AD

**Figure 2.**
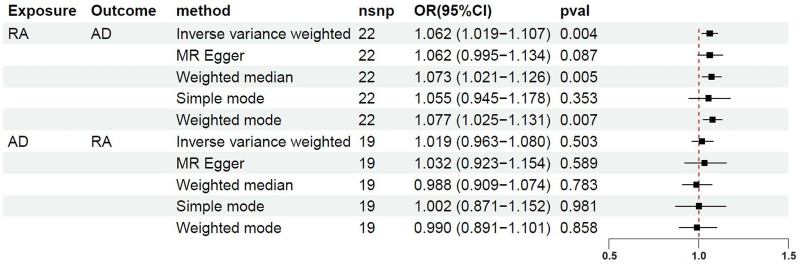
Forest plots of bidirectional Mendelian randomization study on the relationship between RA and AD. RA = rheumatoid arthritis, AD = Alzheimer disease.

**Figure 3.**
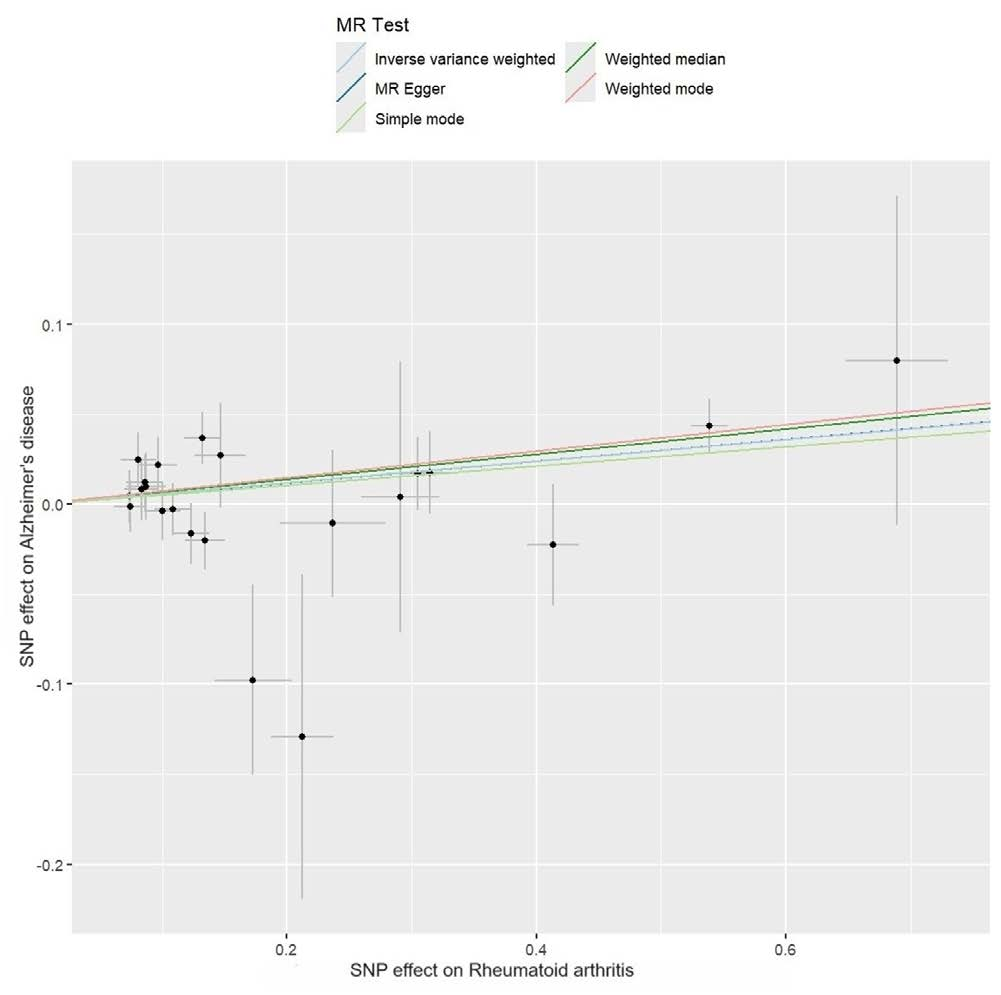
Scatter plots of the causal effect of rheumatoid arthritis on Alzheimer disease.

The IVW method supported a significant positive association between RA and AD (OR = 1.062, 95% CI = 1.019–1.107, *P* = .004). This association was substantiated by the weighted median method (OR = 1.073, 95% CI = 1.021–1.126, *P* = .005) and the weighted mode method (OR = 1.077, 95% CI = 1.025–1.131, *P* = .007). In addition, the OR values of all methods were consistent in terms of direction, thereby reinforcing evidence of the positive causation (Figs. 2 and 3).

**Figure 4.**
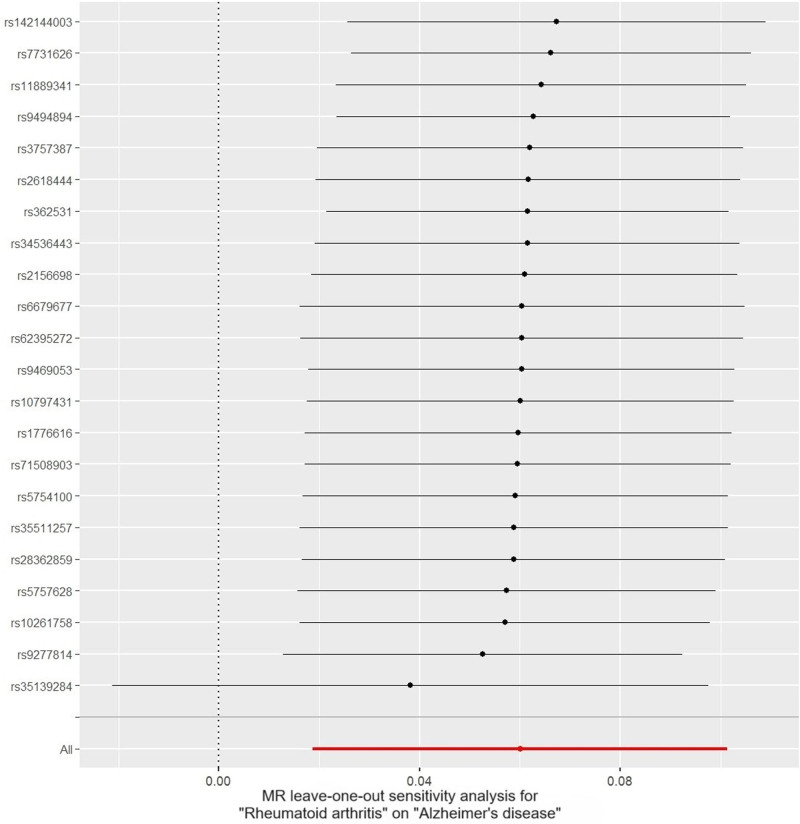
Leave-one-out analysis of the causal effect of rheumatoid arthritis on Alzheimer disease.

**Figure 5.**
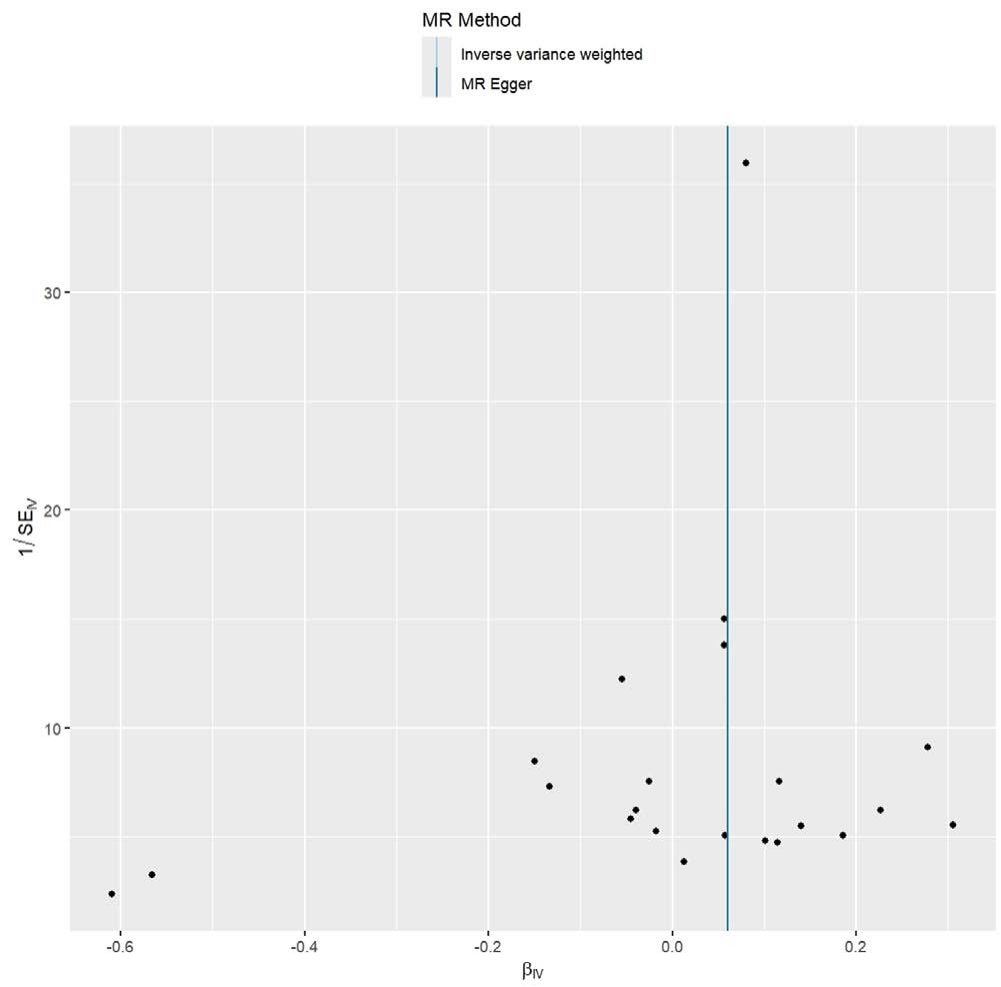
Funnel plots of the causal effect of rheumatoid arthritis on Alzheimer disease.

In the sensitivity analyses, Cochran *Q* test showed no heterogeneity (both IVW and MR Egger *P* > .05), and MR Egger intercept (intercept = ‐7.31E‐05, *P* = .991) and MR-PRESSO global test (*P* = .353) revealed no significant evidence of pleiotropy. Meanwhile, MR-PRESSO analysis did not detect any outliers. Additionally, leave-one-out analysis suggested that the causal association between RA and AD was not driven by any single SNP (Fig. 4). Funnel plots showed that the distribution of the SNPs was symmetrical (Fig. 5).

### 3.3. Casual effect of AD on RA

**Figure 6.**
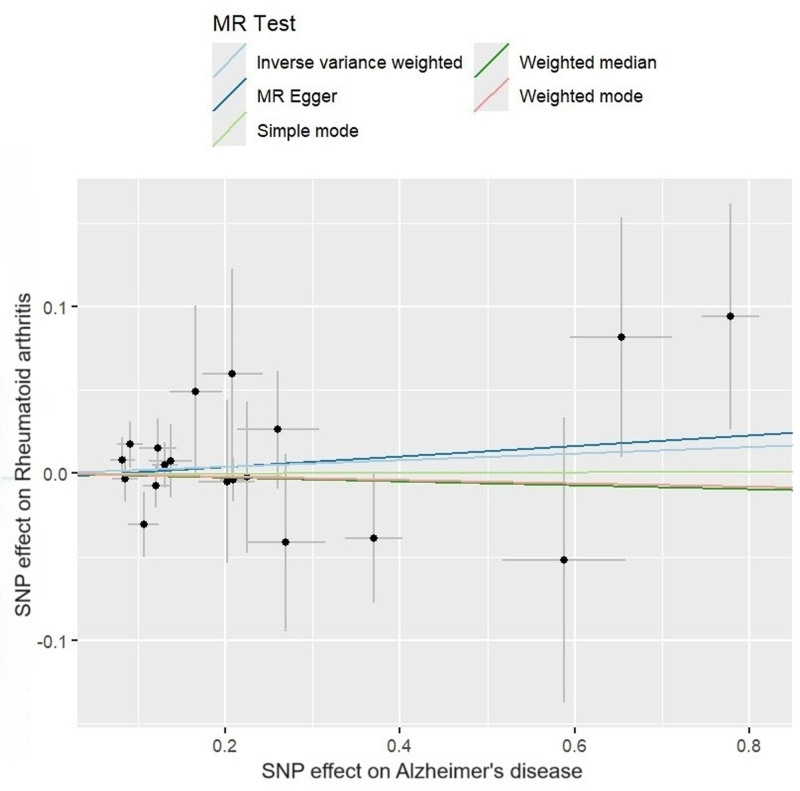
Scatter plots of the causal effect of Alzheimer disease on rheumatoid arthritis.

In the reverse MR analysis, the IVW method did not demonstrate a causal association between genetically predicted AD and the risk of RA (OR = 1.019, 95% CI = 0.963–1.080, *P* = .503). This result received further support from the other 4 MR methods (all *P* > .05) (Figs. 1 and 6).

**Figure 7.**
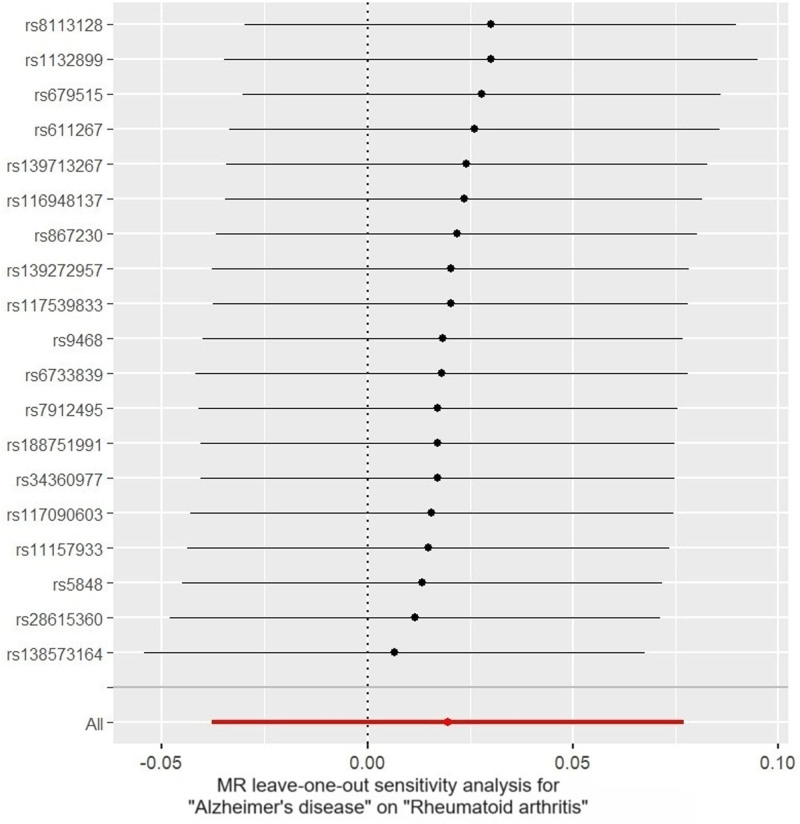
Leave-one-out analysis of the causal effect of Alzheimer disease on rheumatoid arthritis.

**Figure 8.**
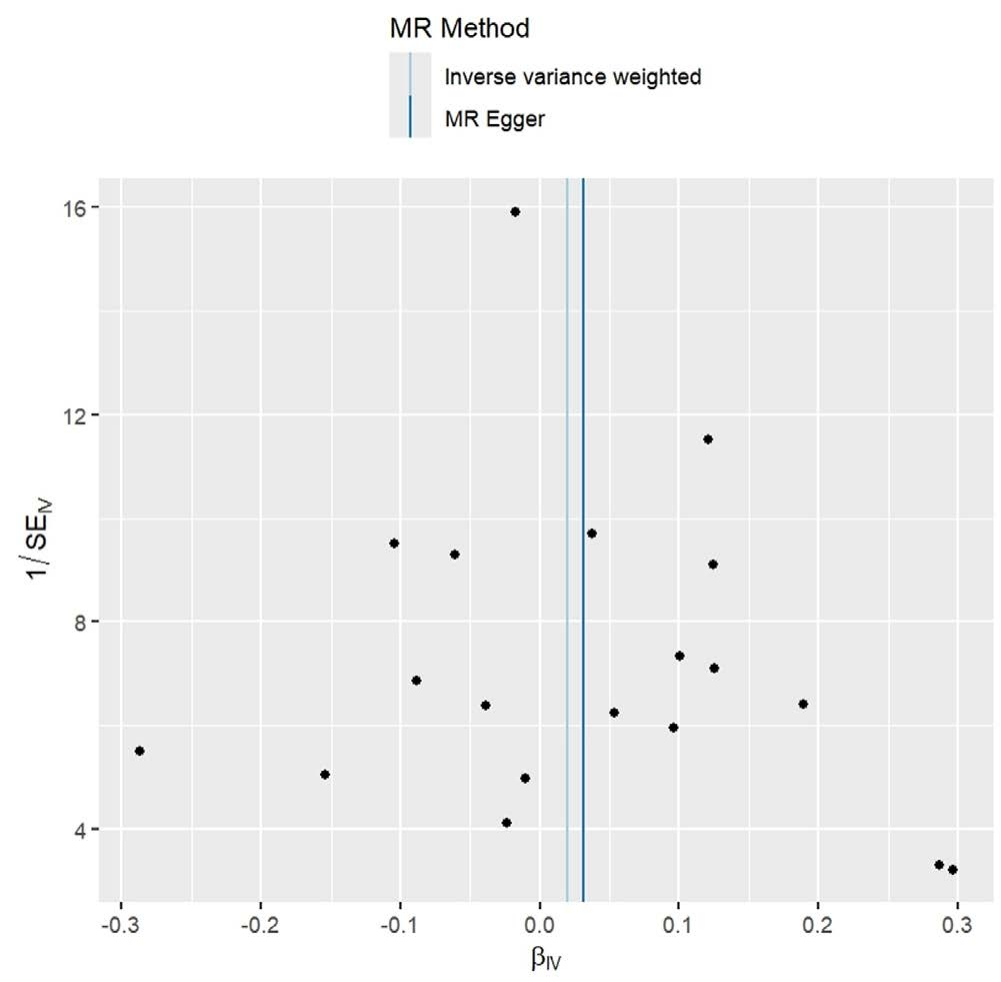
Funnel plots of the causal effect of Alzheimer disease on rheumatoid arthritis.

In the sensitivity analyses, Cochran *Q* test showed no heterogeneity (both IVW and MR Egger *P* > .05), and MR Egger intercept (intercept = ‐0.002, *P* = .812) and MR-PRESSO global test (*P* = .789) revealed no significant evidence of pleiotropy. Meanwhile, MR-PRESSO analysis did not detect any outliers. In addition, leave-one-out analysis indicated that no single SNP significantly influenced the results (Fig. 7). Funnel plots showed that the distribution of the SNPs was symmetrical, demonstrating the robustness of our results (Fig. 8).

## 4. Discussion

Cognitive impairment was relatively prevalent among patients with RA, yet research on the causal relationship between RA and AD remained limited. Based on updated and larger GWAS data, this study explored the causal association between RA and AD in European populations using a 2-sample bidirectional MR method. The results revealed a significant causal relationship between genetically predicted RA and an increased risk of AD. In addition, reverse analysis did not find any correlation between genetic prediction of AD and RA. No pleiotropy or heterogeneity was found in this study, and the robustness and reliability of the findings were confirmed through leave-one-out analysis and funnel plots.

The main findings of this study were consistent with the majority of previous research, and inflammation was recognized as one of the key biological mechanisms underlying the association between RA and AD.^[36–38]^ RA was considered to be a systemic inflammatory disease characterized by a sustained increase in the levels of cytokines such as TNF and interleukin (IL).^[39]^ Persistent systemic inflammation could affect the neurodegenerative processes specific to AD. The activity of inflammatory cytokines (such as TNF-α, IL-1 β, and IL-6) was significantly enhanced in AD patients compared to healthy controls.^[40]^ Given that an overactive immune response was a common feature of both RA and AD, the roles of these cytokines and their involvement in the pathogenesis of AD and RA have become the focus of current research.^[41]^ It was widely believed that the systemic chronic inflammation of RA might disrupt the blood–brain barrier, leading to the leakage of inflammatory factors through the blood–brain barrier into the central nervous system, further activating microglia and astrocytes to trigger neuroinflammation.^[42,43]^ Furthermore, significant progress has been made in exploring anti-inflammatory therapeutic strategies as potential targets for preventing and treating AD. For example, the application of biological agents, such as TNF inhibitors, in the treatment of RA has been proved to reduce the risk of AD.^[23]^ Additionally, experimental data from animal models supported the therapeutic potential of anti-TNF drugs via intrathecal or intracerebroventricular administration, which could improve cognitive function by reducing TNF-α levels and subsequent Aβ deposition.^[44,45]^

Apart from common inflammatory mechanisms, immune abnormalities in RA might affect the risk of AD. Specific biomarkers could be detected in the serum of patients with RA, including anti-cyclic citrullinated peptide, amyloid A, C-reactive protein, and rheumatoid factor.^[46]^ It was worth noting that anti-cyclic citrullinated peptides were not only closely associated with RA, but have also been found to be associated with AD, which might cross recognize brain tissue antigens to attack neurons or glial cells through molecular simulations.^[47]^ In addition, the pathological significance of amyloid protein was reflected in both RA and AD diseases.^[48]^ Amyloid in RA played a role in the deterioration of osteoarthrosis; in AD, on the other hand, Aβ deposition exacerbated neuronal vulnerability, making them more susceptible to excitotoxicity.^[36]^ The association between C-reactive protein and AD risk has also received attention, and further research has revealed a direct link between chronic elevated C-reactive protein levels in RA and an increased risk of later development of AD.^[15]^ At the same time, the deposition and circulation of immune complexes produced by rheumatoid factor might lead to cerebral vasculitis, which could affect cerebral blood flow and cognitive function.^[49]^ Furthermore, peripheral immune cells, such as neutrophils, could reach the brain through the impaired blood–brain barrier, leading to abnormal pathological protein metabolism and neuroinflammation.^[50,51]^

It was well known that RA patients were often accompanied by anxiety, depression, sleep disorders and reduced activity.^[3,5]^ These complications, in turn, were considered as significant factors that exacerbated the risk of developing AD.^[52]^ Chronic pain and physical disability in RA patients could increase their psychological burden, which in turn triggered the hypothalamic–pituitary–adrenal axis to cause a significant increase in cortisol levels, thereby causing damage to hippocampal neurons.^[53]^ In addition, RA patients often experienced insomnia or sleep fragmentation due to pain or side effects of medication, and sleep was a critical period for clearing Aβ in the brain, and sleep deprivation accelerated the pathology of AD.^[54]^ Meanwhile, joint pain and physical fatigue greatly limited movement, while exercise deficiency was associated with decreased brain-derived neurotrophic factor and brain atrophy.^[55]^ Therefore, RA and its accompanying complications posed potential threats to brain health through multiple mechanisms, increasing the risk of developing AD.

The strengths of this study lie in the utilization of updated and larger GWAS data, with all SNPs rigorously screened to ensure the credibility and reliability of the instrumental variables. And MR analysis could minimize errors in the causal estimation between RA and AD caused by reverse causality and confounding factors. Additionally, various analytical methods and sensitivity tests ensured the stability of causal estimates. It should be noted that the data in this study were derived from a European population, and differences among different ethnic groups could not be ruled out, thus limiting the generalizability of the study results. Furthermore, this study only explored the causal relationship between RA and AD, and the specific biological mechanisms still need further experimental research.

## 5. Conclusion

In summary, this study provides genetic evidence supporting a significantly increased risk of AD in patients with RA. This correlation may be the result of the interaction of multiple pathological processes, with the core being neuroinflammation and immune abnormalities driven by chronic inflammation. Furthermore, comorbidities and lifestyle factors further amplify this risk. It is worth noting that the observed effect pertains specifically to individuals of European ancestry, rather than being generalized to all populations. Future research directions include more in-depth exploration of biological mechanisms, larger-scale epidemiological investigation, and development of potential therapeutic targets. Further studies are expected to provide new perspectives and strategies for the prevention and treatment of these 2 diseases.

## Acknowledgments

We are grateful to the foundations mentioned above and all the participants in the study.

## Author contributions

**Data curation:** Xia Liang.

**Methodology:** Shizhan Li.

**Software:** Yanni Lin.

**Validation:** Jianqing Zhu.

**Writing – original draft:** Changqiang Feng, Songxin Zhong.

**Writing – review & editing:** Chao Qin.

## Supplementary Material
